# Clinical and Mycological Profile of Dermatophyte Infections in South‐East England: A 17‐Year Retrospective Analysis (2006–2023)

**DOI:** 10.1111/myc.70165

**Published:** 2026-02-18

**Authors:** Khanh Linh Phan, David J. Chandler

**Affiliations:** ^1^ Department of Global Health & Infection Brighton and Sussex Medical School Brighton UK; ^2^ Dermatology Department University Hospitals Sussex NHS Foundation Trust Brighton UK

## Abstract

**Background:**

Dermatophyte infections, which are among the most prevalent fungal infections globally, affect skin, hair and nails, accounting for significant morbidity. Epidemiological data on dermatophytosis in the UK are limited. One notable study in 2007 provided insights into the causative agents of dermatophyte infections in the UK; however, no extensive regional analysis has been published since.

**Objectives:**

The aim of this study was to provide a contemporary understanding of the epidemiology and causative agents of dermatophytosis in South‐East England.

**Method:**

This retrospective laboratory database review analysed all samples of skin, hair and nail that underwent mycological examination (microscopy and culture) in a single diagnostic microbiology laboratory in South‐East England over a period of 17 years (2006–2023).

**Results:**

Between 2006 and 2023, a total of 34,624 samples were collected, with the majority being nail (*n* = 26,362), followed by skin (*n* = 8015), hair (*n* = 246) and one unknown sample type. Fungal culture yielded positive results in 22.0% (*n* = 7601) of samples, with dermatophytes comprising 89.4% (*n* = 6794) and non‐dermatophyte moulds 4.8% (*n* = 366). Trichophyton species dominated dermatophyte isolates at 99.3% (*n* = 6745), followed by Microsporum (0.5%, *n* = 37) and Epidermophyton (0.2%, *n* = 12). Onychomycosis of toenails was the most common infection (*n* = 2774), predominantly affecting males (median age: 48 years); the cause was *Trichophyton rubrum* in 80.5% of cases, and infection was confirmed by positive direct microscopy in 80.3%. Tinea pedis (*n* = 416), conversely, was more common in females (median age: 50 years). Other dermatophyte infections included tinea corporis (*n* = 179), onychomycosis of the fingernails (*n* = 99), tinea cruris (*n* = 91), tinea capitis (*n* = 82), tinea manuum (*n* = 56) and tinea faciei (*n* = 14). *Trichophyton rubrum* was the primary causative agent across all body sites except for tinea capitis, where *Trichophyton mentagrophytes* (30.5%), *Trichophyton violaceum* (19.5%) and *Trichophyton tonsurans* (15.9%) prevailed. Non‐anthropophilic species (*n* = 165) caused infection across all body sites, but most caused tinea capitis (25.5%) and tinea corporis (20.0%) and affected younger individuals (median age: 29 years). *Trichophyton mentagrophytes* was the most common non‐anthropophilic dermatophyte. Over the 17 years, the prevalence of *Trichophyton interdigitale* declined by 20%, while *Trichophyton rubrum* increased from 78.4% in 2006 to 82.0% in 2023. No cases of *Trichophyton indotineae* were identified.

**Conclusion:**

This study highlights the high prevalence of *Trichophyton rubrum* as a cause of dermatophyte infections in South‐East England. Non‐anthropophilic infections are rare and demographically distinct, guiding case management and public health strategies.

## 1 Introduction

Dermatophyte infections, caused by a range of filamentous fungi, present a significant health concern globally, generating a significant morbidity and economic burden [1, 2]. Between 2005 and 2014, these infections accounted for nearly 4.98 million outpatient visits in the United States, with direct medical costs reaching approximately USD 845 million in 2019, as reported by the Centers for Disease Control and Prevention (CDC) [3, 4].

These infections affect the skin, hair and nails, utilising keratin in epidermis (stratum corneum), hair follicles and nails [5]. Dermatophytes produce enzymes like keratinase, allowing them to break down keratin—a unique feature that distinguishes them from other pathogens and plays a central role in their pathogenicity [6, 7].

Clinically, dermatophyte infections manifest in several forms, such as tinea corporis (ringworm), tinea pedis (athletes' foot), tinea cruris (jock itch), tinea capitis (scalp ringworm) and onychomycosis. Less common presentations include tinea manuum (hand infection) and tinea faciei (facial infection). These infections are characterised by symptoms including pruritus, erythema and scaling, which can significantly impair quality of life [8].

Dermatophytes are classified into three primary genera—*Trichophyton*, *Microsporum* and *Epidermophyton*—and are further grouped based on their ecological niche: anthropophilic (human‐associated), zoophilic (animal‐associated) and geophilic (soil‐associated) [9, 10]. Anthropophilic species, notably *Trichophyton rubrum*, are predominant worldwide, particularly in cases of onychomycosis and tinea corporis [11, 12, 13]. Species such as *Microsporum canis*, which commonly inhabit animals, can be transmitted to humans and frequently cause tinea capitis in children [14, 15]. Geophilic dermatophytes, like *Microsporum gypseum*, although less common, are responsible for infections following direct contact with soil or organic material [9, 16].

Effective management of dermatophyte infections and public health efforts requires an understanding of local and global epidemiology. Despite growing research activity in recent years, knowledge gaps remain, especially at a regional level. Ortiz et al. highlighted the need for updated region‐specific data on species prevalence and emerging antifungal resistance in their bibliometric analysis [17]. Studies have shown shifts in dermatophyte species distribution, with anthropophilic species such as *Trichophyton indotineae* becoming increasingly common in many countries throughout Asia and Europe [18]. These findings underscore the need for updated surveillance in regions like the United Kingdom (UK), where the last comprehensive review on the causative agents of dermatophytosis was conducted by Borman et al. in 2007.

In this study, *Trichophyton rubrum* and *Trichophyton interdigitale* were the dominant species, responsible for most cases of onychomycosis and tinea pedis, while *Microsporum canis* infections, especially among children, were linked to the prevalence of pets as reservoirs in the UK^19^. However, shifts in dermatophyte ecology and public health changes in the past two decades may have influenced species prevalence. This is supported by recent epidemiological data from Switzerland and Ireland, which suggest a rise in infections caused by less common dermatophytes, along with changing antifungal resistance patterns [19, 20, 21, 22]. Contemporary data are needed to provide an updated understanding of dermatophytosis, particularly in the UK, where data are lacking.

## 2 Materials and Methods

This study utilised data provided by the Microbiology & Infection department at University Hospitals Sussex (East), which serves as the main microbiology centre for healthcare institutions throughout East Sussex, including hospitals, general practitioner (GP) clinics, outpatient clinics and community healthcare services.

Laboratory data between 2006 and 2023 were extracted from the WinPath Laboratory Information Management System (LIMS) using the PathManager reporting tool (Clinisys, UK). Mycological assessment initially involved potassium hydroxide (KOH) microscopy, with Calcofluor white (CFW) fluorescence microscopy introduced in 2018 as part of routine laboratory practice. Cultures were cultivated on Sabouraud agar (SDA) plates containing chloramphenicol and actidione for a two‐week incubation period at 30°C. Dermatophyte isolates were identified based on their macroscopic and microscopic features of the fungi.

In samples yielding mixed growth, dermatophytes were considered clinically significant when isolated in culture, irrespective of the presence of concomitant non‐dermatophyte moulds or yeast. Non‐dermatophyte organisms identified in mixed cultures were recorded but were not considered primary pathogens unless specifically indicated by the requesting clinician. In the absence of clinical correlation, repeat sampling or histopathological confirmation, non‐dermatophyte mould isolates were not classified as definitive causes of onychomycosis and were reported descriptively.

Yeast was identified using matrix‐assisted laser desorption ionisation‐time of flight (MALDI‐TOF) mass spectrometry or Auxacolour biochemical kit (Bio‐Rad Laboratories, California). Our analysis focused exclusively on skin, hair and nail specimens with data processed using Microsoft Excel. Temporal changes in species distribution were assessed by comparing the early (2006–2010) and late (2019–2023) study periods using χ^2^ test (2 × 2). A *p*‐value of < 0.05 was considered statistically significant. Difference in sex distribution across infection sites (skin, hair and nail) was examined using a χ^2^ test (3 × 2), excluding cases with missing sex data. Differences in sex distribution between tinea pedis and other skin dermatophyte infections (excluding hair and nail) were assessed using a two‐proportion z‐test.

The clinical condition was determined based on the specific anatomical sites documented by the sample collector, categorised as listed below:

1. Tinea capitis: skin and hair samples from the scalp, hairline and head
2. Tinea faciei: skin samples from the face, eyelid
3. Tinea corporis: skin samples from the abdomen, arm, wrist, back, chest, thigh, trunk, neck and ears
4. Tinea manuum: skin samples from the hand, finger, palm, thumb
5. Tinea pedis: skin samples from the feet, toes, ankle and toe web spaces
6. Onychomycosis of the fingers: fingernail, thumbnail
7. Onychomycosis of the toe: nail samples under the toe, little toe, big toe
8. Tinea cruris: skin samples taken from areas such as the buttocks, genitalia, groin, inner thighs, labia, perineum, suprapubic region and vulva

This project received governance approval within University Hospitals Sussex NHS Foundation Trust to be conducted as a service evaluation (unique identifier: 2516).

## 3 Results

**FIGURE 1:**
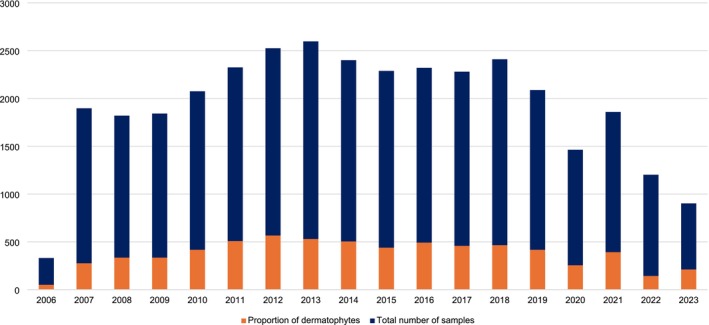
Annual number of dermatophytes isolated as a proportion of all samples received (2006*–*2023). The figure illustrates the yearly proportion of dermatophyte‐positive cultures relative to the total number of samples processed between 2006 and 2023, demonstrating temporal trends in dermatophyte isolation rates.

Between 2006 and 2023, a total of 34,624 samples were received. Out of these, 22.0% (*n* = 7601) had a positive culture for mould and/or yeast, while 68.9% (*n* = 23,841) had no fungal growth after 14 days. 9.1% (*n* = 3182) of samples could not be processed for reasons such as insufficient clinical material or labelling errors. Samples yielding dermatophytes, as a proportion of all samples received, are shown for each year in the study period (Figure 1).

Regarding the types of specimens, nail samples accounted for 76.1% (*n* = 26,362), skin samples for 23.1% (*n* = 8015), hair samples for 0.7% (*n* = 246) and there was one unknown sample.

Most samples were received from GP clinics (84.4%, *n* = 29,221). Outpatient clinics contributed to 11.6% (*n* = 4027) of the samples, with a significant portion (83.7%) being from dermatology‐specific clinics. Community services represented 1.1% (*n* = 396) of the samples, and hospitals accounted for 1.6% (*n* = 561), with half of the hospital samples being collected from inpatient settings. A small proportion of samples (1.2%, *n* = 419) had an unknown collection location.

### 3.1 Positive Culture—Dermatophytes

Analysis of the positive cultures revealed that 89.4% (*n* = 6794) showed growth of dermatophytes. *Trichophyton* species were predominant among dermatophyte isolates, with 6745 samples identified as *Trichophyton*, followed by 37 samples of *Microsporum* and 12 samples of *Epidermophyton*.

Within the *Trichophyton* group, the vast majority of 99.4% (*n* = 6704) consisted of pure *Trichophyton* isolates, while 0.6% (*n* = 41) displayed mixed growth with both dermatophyte and non‐dermatophyte organisms. In cases of mixed growth, the dermatophyte isolate was regarded as the causative organism. In cases of mixed growth with *Trichophyton*, *Candida* species were the most observed non‐dermatophytes in over half of the samples (*n* = 24). *Trichophyton rubrum*, together with *Candida*, was commonly seen in 19 samples; among these, toenails accounted for nine cases, while eight were unspecified nail sample sites and one each from fingernail and breast sites. Five samples grew *Trichophyton interdigitale* together with *Candida*. Three were from the toenails and two nail samples with unidentified sites.

### 3.2 Positive Culture—Non‐Dermatophyte Moulds

**FIGURE 2:**
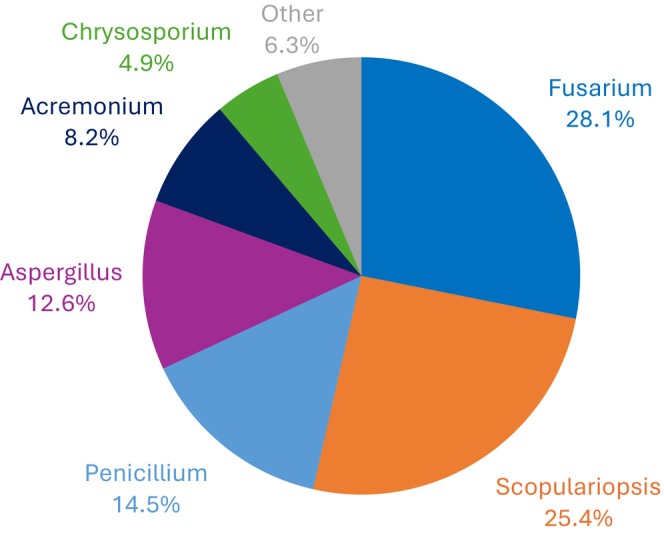
Distribution of non‐dermatophyte moulds. The figure shows the relative frequency of non‐dermatophyte mould species isolated during the study period, expressed as a percentage of total non‐dermatophyte isolates.

Non‐dermatophyte moulds were detected in 4.8% (*n* = 366) of cases, with *Fusarium* comprising the majority at 28.1% (*n* = 103), followed by *Scopulariopsis* at 25.4% (*n* = 93), *Penicillium* at 14.5% (*n* = 53), *Aspergillus* at 12.6% (*n* = 46), *Acremonium* at 8.2% (*n* = 30) and *Chrysosporium* at 4.9% (*n* = 18). Other species, like *Exophiala, Rhodotorula, Chaetomium* and *Neoscytalidium*, collectively made up for 6.3% (*n* = 23) and were grouped under “Other.” These findings are visually represented in (Figure 2).

### 3.3 Positive Culture—Other

Further analysis of positive cultures revealed the presence of yeast growth in 6.4% (*n* = 487) of cultured isolates. Among these cases, *Candida* species accounted for the majority with a total of 483 isolates identified; two isolates were classified as *Malassezia*, while two others belonged to an unidentified yeast genus. Additionally, in one sample, the dimorphic pathogen *Sporothrix schenckii* was isolated. Bacterial growth was also noted in eight samples: seven *Pseudomonas* species and one *Rhodococcus* species.

### 3.4 Direct Microscopy Results

Microscopy findings were reported alongside culture results. Of the 6794 samples that displayed dermatophyte growth, 76.6% (*n* = 5207) had positive microscopy, demonstrating the presence of fungal hyphal elements, whilst 22.0% (*n* = 1495) were negative. Notably, 1.4% (*n* = 92) of the dermatophyte samples did not undergo microscopy due to reasons including insufficient sample quantity, unlabelled specimens or samples deemed unsuitable for microscopic examination.

Onychomycosis of the toes and fingers exhibited the highest percentage of positive microscopy results at 80.3% and 74.8%, respectively (Table 1). In contrast, tinea capitis had the lowest positivity rate at 24.4%.

**TABLE 1:** Frequency of positive versus negative microscopy results in confirmed dermatophytosis (positive culture) across the different anatomical sites.

	Hair	Skin							Nail			
	Tinea capitis	Tinea faciei	Tinea corporis	Tinea manuum	Tinea pedis	Tinea manuum + pedis	Tinea cruris	Tinea of unknown site	Onychomycosis finger	Onychomycosis toe	Onychomycosis of unknown site	Total number of samples
Number of infections (positive culture), *n*	82	14	179	56	416	2	91	149	99	2774	2932	6794
Microscopy positive, *n* (%)	20 (24.4)	8 (57.1)	89 (49.7)	33 (58.9)	234 (56.2)	1 (50.0)	37 (40.7)	70 (47.0)	74 **(74.8)**	2228 **(80.3)**	2413 **(82.3)**	5207 (76.6)
Microscopy negative, *n* (%)	49 (59.8)	6 (42.9)	76 (42.5)	16 (28.6)	160 (38.5)	1 (50.0)	45 (49.4)	71 (47.6)	23 (23.2)	535 (19.3)	513 (17.5)	1495 (22.0)
Not tested, *n* (%)	13 (15.8)	0 (0.0)	14 (7.8)	7 (12.5)	22 (5.3)	0 (0.0)	9 (9.9)	8 (5.4)	2 (2.0)	11 (0.4)	6 (0.2)	92 (1.4)

*Note:* Microscopy was performed using potassium hydroxide (KOH) wet mount preparation. A result was considered positive when fungal hyphae or spores were visualised microscopically.

Annual dermatophyte‐positive microscopy rates varied throughout the study period. During 2006 and 2018, when KOH microscopy was used, the mean positivity rate was 74.7%. Following the introduction of CFW fluorescence microscopy in 2018, the mean positivity rate increased to 79.5% during 2018–2023. Year‐to‐year fluctuations were observed in both periods, without a consistent temporal trend.

### 3.5 Dermatophytosis—Clinical and Demographic Data

Out of 6794 dermatophyte isolates, 3713 had a recorded anatomical site. Among these, onychomycosis was the predominant infection, accounting for 2873 cases (77.4%). Most onychomycosis cases originated from toenails (*n* = 2774; 96.6%), while a minority were from fingernails (*n* = 99). Cutaneous infections represented 840 cases, with tinea pedis being the most frequent (*n* = 416), followed by tinea corporis (*n* = 179). Other infections, including tinea cruris (*n* = 91), tinea capitis (*n* = 82), tinea manuum (*n* = 56) and tinea pedis (*n* = 14), were observed but were less common.

Of note, 3081 isolates lacked site information. Within this group, 95.2% (*n* = 2932) were identified as nail samples; however, it was not possible to distinguish whether these were of finger or toe origin.

Nail and hair specimens were mostly collected from female patients (56.3% female vs. 43.7% male), unlike skin specimens, which had a more balanced gender distribution (48.4% female vs. 51.6% male). Dermatophyte infections were generally more prevalent in males than females. When sex distribution was compared across anatomical categories (skin, hair and nail), no significant differences were observed (χ^2^ = 3.96, df = 2, *p* = 0.14). However, within skin infections specifically, tinea pedis showed a different pattern; females slightly outnumbered males (52.5% vs. 47.5%). When formally compared, the proportion of male cases was significantly lower in tinea pedis than in all other skin dermatophyte infections combined (47.5% vs. 63.8%; z = 4.69, *p* < 0.001).

Analysis of patient age according to the anatomic site of infection is shown in (Table 2). Dermatophyte infections typically affected individuals between the ages of 47 and 53 years across all sites, except for tinea capitis, which predominantly affected younger individuals (median age of 5 years). Tinea faciei was found to be more common in young adults (median age: 24 years).

**TABLE 2:** Distribution of dermatophyte infections by anatomical site.

	Hair	Skin						Nail			
	Tinea capitis	Tinea faciei	Tinea corporis	Tinea manuum	Tinea pedis	Tinea cruris	Tinea of unknown site	Onychomycosis finger	Onychomycosis toe	Onychomycosis of unknown site	Total number
Sex distribution											
Number of samples, *n*	78	14	178	55	413	90	145	99	2755	2919	6746
% Female	46.2	28.6	42.7	27.3	52.5	25.8	38.6	24.2	41.4	40.7	41.2
% Male	53.8	71.4	57.3	72.7	47.5	74.2	61.4	**75.8**	58.6	59.3	58.8
Age (years)											
Number of samples, *n*	70	14	169	51	400	87	144	96	2763	2925	6719
Median	5	24	53	47	50	49	48	51	48	47	47
IQR	4–8	8–52	37–68	31–58	39–66	40–66	33–64	36–64	35–62	34–60	34–61

*Note:* Data represent the number and percentage of culture‐confirmed dermatophyte infections identified at each anatomical site. Percentages are calculated based on the total number of dermatophyte isolates.

Abbreviations: *n*, number of isolates; %, percentage.

### 3.6 Causative Agents of Dermatophytosis by Anatomical Site

**FIGURE 3:**
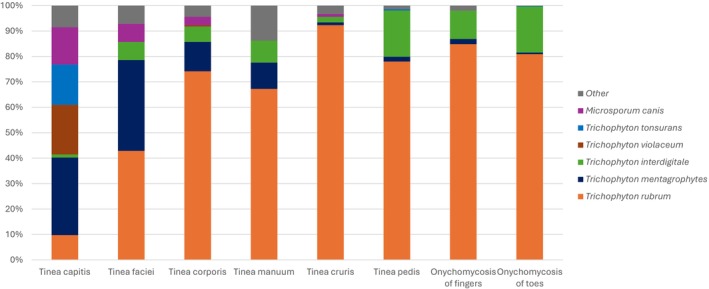
Distribution of dermatophyte species across different anatomical sites. The figure illustrates the distribution of major dermatophyte species isolated from various anatomical sites, including *Trichophyton rubrum*, *T. mentagrophytes*, *T. interdigitale*, *T. violaceum* , *T. tonsurans* and *Microsporum canis*. “Others” represents the remaining dermatophyte species identified in the study.

*Trichophyton rubrum* was identified as the most common dermatophyte, accounting for over 70% of cases of tinea pedis, tinea corporis, onychomycosis of the fingers and toes, and tinea cruris (as illustrated in Figure 3). However, in instances of tinea capitis, *Trichophyton rubrum* constituted less than 10% of isolates. The main causative agents of tinea capitis were *Trichophyton mentagrophytes* (30.1%), *Trichophyton violaceum* (19.3%), *Trichophyton tonsurans* (15.7%) and *Microsporum canis* (14.5%). For tinea faciei, *Trichophyton rubrum* and *Trichophyton mentagrophytes* accounted for 42.9% and 35.7% of cases, respectively.

### 3.7 Causative Agents of Dermatophytosis—Trends Over Time

Small shifts in the prevalence of dermatophyte species were observed over the 17 year study period. The total number of isolates for each species is summarised in (Table 3), while temporal changes in their relative proportions are shown in (Table 4). *Trichophyton rubrum* was the most common species across all years. At the beginning of the study (2006), *T. rubrum* accounted for 78.4% of dermatophytes, followed by *T. interdigitale* (15.7%), *T. mentagrophytes* (2.0%), *T. tonsurans* (2.0%) and *M. canis* (2.0%). By 2009, the prevalence of *T. interdigitale* had increased to 24.8%, *T. rubrum* had decreased to *71.3%*, whereas *T. tonsurans* and *T. mentagrophytes* contributed less than 3% combined. In 2021, towards the end of the study period, the prevalence of *Trichophyton rubrum* peaked at 85.7%.

**TABLE 3:** Dermatophytes isolated from 2006 through 2023, expressed as total numbers of isolates.

	A. ben	E. flocc	M. aud	M. canis	M. fulv	M. gyps	M. persi	Microsporum species	T. ben	T. equi	T. erin	T. int	T. ment	T. pers	T. rubr	T. soud	T. terr	T. tons	T. verr	T. viol	Trichophyton species	Total
2006	0	0	0	1	0	0	0	0	0	0	0	8	1	0	40	0	0	1	0	0	0	51
2007	0	1	0	0	0	0	0	0	0	0	0	38	4	0	220	0	0	13	0	1	0	277
2008	0	0	0	1	0	0	0	0	0	0	1	64	10	0	257	0	2	1	0	1	0	337
2009	0	0	0	0	0	1	0	0	0	0	0	83	4	0	239	0	0	3	1	3	1	335
2010	0	0	1	1	0	1	0	0	0	0	1	57	7	0	344	0	1	1	0	3	0	417
2011	0	0	0	1	0	0	0	1	0	0	1	74	6	1	423	0	0	0	0	2	0	509
2012	0	2	0	3	0	0	0	0	0	0	0	105	9	0	448	0	0	1	0	2	0	570
2013	0	2	0	0	0	1	0	0	0	0	0	89	21	0	427	0	0	1	0	2	0	543
2014	0	1	1	2	0	0	1	0	0	0	0	83	14	0	403	0	0	0	0	0	0	505
2015	0	0	0	2	0	0	0	0	0	0	1	68	8	0	356	1	1	1	0	1	0	439
2016	0	1	0	1	0	0	0	0	0	0	0	66	4	0	417	0	1	0	0	3	0	493
2017	0	2	1	1	0	0	0	0	0	0	1	84	3	0	365	0	0	0	0	1	0	458
2018	0	1	0	0	0	2	0	0	0	0	0	85	1	0	373	1	0	2	0	0	0	465
2019	0	0	0	1	2	0	0	0	0	0	0	75	1	0	337	0	0	0	0	1	0	417
2020	0	0	0	3	0	1	0	0	1	2	0	26	7	0	217	0	0	0	0	0	0	257
2021	0	0	0	0	0	0	0	0	0	0	1	48	6	0	336	0	0	1	0	0	0	392
2022	1	1	0	0	0	1	0	0	0	0	0	15	7	0	119	0	0	0	0	0	0	144
2023	0	1	0	6	0	0	0	0	0	0	2	26	3	0	173	0	0	0	0	0	0	211
Total	1	12	3	23	2	7	1	1	1	2	8	1094	116	1	5494	2	5	25	1	20	1	6820

*Note:* Data represent the total number of dermatophyte isolates identified during the study period. A. ben, A. benhamiae; E. flocc, E. floccosum; M. aud, M. audouinii; M. canis, *M. canis* ; M. gyps, M. gypseum; M. pers., M. persicolor; T. ben, T. benhamiae; T. equi, T. equinum; T. erin, T. erinacei; T. int., T. interdigitale; T. ment, T. mentagrophytes; T. pers., T. persicolor; T. rubr., *T. rubrum* ; T. soud, T. soudanense; T. terre, T. terrestre; T. tons, T. tonsurans; T. verr, *T. verrucosum* ; T. viol, T. violaceum. Columns labelled “Microsporum Species” and “Trichophyton Species” are samples with unspecified species name.

**TABLE 4:** Dermatophytes isolated from 2006 through 2023, expressed as percentage of total isolates.

	A. ben	E. flocc	M. aud	M. canis	M. fulv	M. gyps	M. persi	Microsporum species	T. ben	T. equi	T. erin	T. int	T. ment	T. pers	T. rubr	T. soud	T. terr	T. tons	T. verr	T. viol	Trichophyton species
2006	0.0%	0.0%	0.0%	2.0%	0.0%	0.0%	0.0%	0.0%	0.0%	0.0%	0.0%	15.7%	2.0%	0.0%	78.4%	0.0%	0.0%	2.0%	0.0%	0.0%	0.00%
2007	0.0%	0.4%	0.0%	0.0%	0.0%	0.0%	0.0%	0.0%	0.0%	0.0%	0.0%	13.7%	1.4%	0.0%	79.4%	0.0%	0.0%	4.7%	0.0%	0.4%	0.00%
2008	0.0%	0.0%	0.0%	0.3%	0.0%	0.0%	0.0%	0.0%	0.0%	0.0%	0.3%	19.0%	3.0%	0.0%	76.3%	0.0%	0.6%	0.3%	0.0%	0.3%	0.00%
2009	0.0%	0.0%	0.0%	0.0%	0.0%	0.3%	0.0%	0.0%	0.0%	0.0%	0.0%	24.8%	1.2%	0.0%	71.3%	0.0%	0.0%	0.9%	0.3%	0.9%	0.30%
2010	0.0%	0.0%	0.2%	0.2%	0.0%	0.2%	0.0%	0.0%	0.0%	0.0%	0.2%	13.7%	1.7%	0.0%	82.5%	0.0%	0.2%	0.2%	0.0%	0.7%	0.00%
2011	0.0%	0.0%	0.0%	0.2%	0.0%	0.0%	0.0%	0.2%	0.0%	0.0%	0.2%	14.5%	1.2%	0.2%	83.1%	0.0%	0.0%	0.0%	0.0%	0.4%	0.00%
2012	0.0%	0.4%	0.0%	0.5%	0.0%	0.0%	0.0%	0.0%	0.0%	0.0%	0.0%	18.4%	1.6%	0.0%	78.6%	0.0%	0.0%	0.2%	0.0%	0.4%	0.00%
2013	0.0%	0.4%	0.0%	0.0%	0.0%	0.2%	0.0%	0.0%	0.0%	0.0%	0.0%	16.4%	3.9%	0.0%	78.6%	0.0%	0.0%	0.2%	0.0%	0.4%	0.00%
2014	0.0%	0.2%	0.2%	0.4%	0.0%	0.0%	0.2%	0.0%	0.0%	0.0%	0.0%	16.4%	2.8%	0.0%	79.8%	0.0%	0.0%	0.0%	0.0%	0.0%	0.00%
2015	0.0%	0.0%	0.0%	0.5%	0.0%	0.0%	0.0%	0.0%	0.0%	0.0%	0.2%	15.5%	1.8%	0.0%	81.1%	0.2%	0.2%	0.2%	0.0%	0.2%	0.00%
2016	0.0%	0.2%	0.0%	0.2%	0.0%	0.0%	0.0%	0.0%	0.0%	0.0%	0.0%	13.4%	0.8%	0.0%	84.6%	0.0%	0.2%	0.0%	0.0%	0.6%	0.00%
2017	0.0%	0.4%	0.2%	0.2%	0.0%	0.0%	0.0%	0.0%	0.0%	0.0%	0.2%	18.3%	0.7%	0.0%	79.7%	0.0%	0.0%	0.0%	0.0%	0.2%	0.00%
2018	0.0%	0.2%	0.0%	0.0%	0.0%	0.4%	0.0%	0.0%	0.0%	0.0%	0.0%	18.3%	0.2%	0.0%	80.2%	0.2%	0.0%	0.4%	0.0%	0.0%	0.00%
2019	0.0%	0.0%	0.0%	0.2%	0.5%	0.0%	0.0%	0.0%	0.0%	0.0%	0.0%	18.0%	0.2%	0.0%	80.8%	0.0%	0.0%	0.0%	0.0%	0.2%	0.00%
2020	0.0%	0.0%	0.0%	1.2%	0.0%	0.4%	0.0%	0.0%	0.4%	0.8%	0.0%	10.1%	2.7%	0.0%	84.4%	0.0%	0.0%	0.0%	0.0%	0.0%	0.00%
2021	0.0%	0.0%	0.0%	0.0%	0.0%	0.0%	0.0%	0.0%	0.0%	0.0%	0.3%	12.2%	1.5%	0.0%	**85.7%**	0.0%	0.0%	0.3%	0.0%	0.0%	0.00%
2022	0.7%	0.7%	0.0%	0.0%	0.0%	0.7%	0.0%	0.0%	0.0%	0.0%	0.0%	10.4%	4.9%	0.0%	82.6%	0.0%	0.0%	0.0%	0.0%	0.0%	0.00%
2023	0.0%	0.5%	0.0%	2.8%	0.0%	0.0%	0.0%	0.0%	0.0%	0.0%	0.9%	12.3%	1.4%	0.0%	82.0%	0.0%	0.0%	0.0%	0.0%	0.0%	0.00%

*Note:* Data represent the percentage of each dermatophyte species relative to the total number of isolates identified during the study period. A. ben, A. benhamiae; E. flocc, E. floccosum; M. aud, M. audouinii; M. canis, *M. canis* ; M. gyps, M. gypseum; M. pers., M. persicolor; T. ben, T. benhamiae; T. equi, T. equinum; T. erin, T. erinacei; T. int., T. interdigitale; T. ment, T. mentagrophytes; T. pers., T. persicolor; T. rubr., *T. rubrum* ; T. soud, T. soudanense; T. terre, T. terrestre; T. tons, T. tonsurans; T. verr, *T. verrucosum* ; T. viol, T. violaceum. Columns labelled “Microsporum Species” and “Trichophyton Species” are samples with unspecified species name.

To formally evaluate these observations, species proportions in the early (2006–2010) and late (2019–2023) study periods were compared using the χ^2^ test. The proportion of isolates identified as *T. rubrum* increased significantly from 77.6% to 83.2% (χ^2^ = 13.5, *p* = 0.0002), whereas the proportion of T. interdigitale decreased significantly from 17.6% to 13.4% (χ^2^ = 9.6, *p* = 0.0019).

Throughout the study period, *Trichophyton interdigitale* consistently ranked as the second most prevalent species, with a prevalence of 12.3% in 2023, slightly lower than its average prevalence of 15.6%. By 2023, all other species had a prevalence below 3%, with some species even absent from the samples. Regrettably, the isolations of other dermatophytes within this dataset, including *Arthroderma benhamiae, Epidermophyton floccosum, Microsporum audouinii, Microsporum fulvum, Microsporum gypseum, Microsporum persicolor, Trichophyton benhamiae, Trichophyton equinum, Trichophyton erinacei, Trichophyton persicolor, Trichophyton soudanense, Trichophyton terrestre, Trichophyton verrucosum* and *Trichophyton violaceum*, were insufficiently low to yield statistically significant trends in prevalence over the specified period.

### 3.8 Causative Agents of Dermatophytosis—Anthropophilic vs. Non‐Anthropophilic Species

Non‐anthropophilic species accounted for 165 (2.4%) of the dermatophytes isolated in this study. These species included: *Arthroderma benhamiae*, *Microsporum canis*, *Microsporum fulvum, Microsporum gypseum, Microsporum persicolor*, *Trichophyton equinum*, *Trichophyton erinacei*, *Trichophyton mentagrophytes* and *Trichophyton verrucosum*.

In these cases, the average patient age was 29 (IQR of 8–52) years. Additionally, there was an approximately equal distribution between females and males. *Trichophyton mentagrophytes* emerged as the predominant isolate, accounting for 70.3% of the identified cases, underscoring its significance in the non‐anthropophilic group.

**FIGURE 4:**
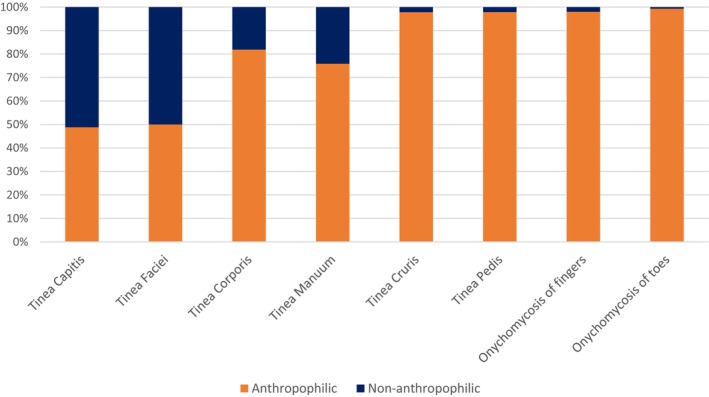
Anthropophilic vs. non‐anthropophilic species causing dermatophyte infection across different anatomical sites. The figure compares the distribution of anthropophilic and non‐anthropophilic dermatophyte species isolated from different anatomical sites.

Comparative analysis with anthropophilic species, as depicted in (Figure 4), reveals distinct patterns. Anthropophilic species consistently dominated, comprising at least 70% of infections. This is particularly evident in onychomycosis cases affecting toenails (99.3%) and fingernails (98.0%), as shown in the right two bars. Conversely, a more even distribution is noted between anthropophilic and non‐anthropophilic species in tinea capitis and tinea faciei, shown in the left two bars. In cases of tinea corporis and tinea manuum, the non‐anthropophilic species accounted for about a quarter of the samples.

No clinical data regarding animal exposure were available for cases classified as non‐anthropophilic. Classification was therefore based on established species ecology rather than documented zoonotic contact.

## 4 Discussion

This study represents the largest analysis of mycological data pertaining to dermatophyte infections in the UK since the study by Borman et al. in 2007 [19].

Our analysis confirms findings that *Trichophyton* species remain the most common cause of dermatophytosis in the UK, with *T. rubrum* being the most isolated species across most body sites. These results are consistent with those reported by Powell et al., who identified *Trichophyton rubrum* as the leading cause of onychomycosis in Ireland between 2001 and 2020 [21]. Similarly, international studies from countries like Switzerland and Korea have also recognised *Trichophyton rubrum* as a predominant cause of dermatophytosis [20, 23]. However, research conducted in Saudi Arabia and various parts of Southern and Eastern Africa has shown a higher proportion of non‐anthropophilic species, reflecting regional differences in dermatophyte ecology and environmental influences [24, 25]. These variations are likely due to factors such as arid climates found in certain areas that may favour the growth of specific non‐anthropophilic over anthropophilic species [26]. Regional differences in hygiene practices, footwear choices and living conditions may also contribute to variations in the range of dermatophyte species present [27, 28]. While current studies provide insights, further research is needed to understand how ecological and environmental factors impact the spread of dermatophytes for effective prevention and treatment strategies.

Although our study did not identify significant trends in the prevalence of less common dermatophyte species like *Trichophyton violaceum* and *Microsporum audouinii*, their occasional detection emphasises the importance of continuous surveillance to monitor fungal populations and identify emerging pathogens.

Beyond species trends, sex distribution also varied by clinical presentation. Although sex proportions did not differ significantly across skin, hair and nail overall, tinea pedis occurred significantly less frequently in males than other skin infections. This suggests that host‐ and site‐specific behavioural or environmental factors may contribute to the observed pattern.

The high rate of positive microscopy results in toenail onychomycosis warrants further discussion. Our findings indicate that 80.3% of samples showed evidence of fungal hyphae presence. This contrasts with studies reporting lower rates of positive microscopy in cases of onychomycosis [29]. The high sensitivity of microscopy in detecting fungal elements in nail samples in our study may be influenced by factors such as sample preparation methods, staining techniques and the expertise of laboratory staff. One potential reason for the higher positivity rate in toenail samples could be due to variations in sample size and hygiene practices. Toenail samples are typically larger than skin scrapings, allowing more material for analysis. However, it is important to acknowledge the limitations associated with microscopy, including false‐positive results and interpretation variability. Further research that combines microscopy with techniques such as polymerase chain reaction (PCR) is necessary to confirm our findings and enhance diagnostic precision [30].

Interpretation of microscopy positivity over time should take into account the change in laboratory technique during the study period. In 2018, routine direct microscopy transitioned from KOH preparation to CFW fluorescence microscopy, resulting in an increased positive direct microscopy rate. CFW enhances visualisation of fungal cell wall components and has been shown to improve detection of fungal elements, particularly in samples with low fungal burden.

The fluctuating prevalence of dermatophytes in 2020 and 2022, followed by a resurgence in 2023, raises points for discussion. While variations in dermatophytosis incidence rates are not uncommon, a quarter of mycology samples in 2022 were left unprocessed by the lab due to COVID‐19‐related workload pressures. Changes in healthcare utilisation patterns and restricted access to facilities during that period may have also influenced these trends. The subsequent increase in dermatophyte prevalence post‐pandemic in 2023 could be linked to the return of healthcare services.

The strengths of our study lie in several key aspects. First, the dataset covers 17 years, providing valuable insights into the epidemiology of dermatophyte infections in England. The wide geographical scope of our study area makes our findings more relevant to local populations and clinical practice. Moreover, our analysis includes information from healthcare facilities, including GP clinics, outpatient clinics and hospitals. This comprehensive approach encompasses a range of demographics and addresses different types of skin infections caused by dermatophytes in medical settings. Furthermore, the incorporation of microscopic methods for detecting fungi enhances the accuracy and sensitivity of our research. By utilising these techniques, we aim to enhance the identification of dermatophyte species, minimising the risk of false‐negative results and offering a more comprehensive understanding of the epidemiological landscape of dermatophyte infections [31].

A limitation of this study is the lack of clinical details, including outcomes following treatment, and the inability to identify *T. indotineae* using the routine methods employed in this diagnostic laboratory, meaning that cases may not have been identified. Confirmed *T. indotineae* infection has been observed in this study catchment area within recent months (data not published). Interpretation of mixed cultures was further limited by the absence of detailed clinical correlation and by routine microscopy reporting, which records the presence of hyphae and/or yeast but does not distinguish dermatophyte from non‐dermatophyte hyphae; similarly, the pathogenic significance of non‐dermatophyte mould isolates, particularly in onychomycosis, could not be confirmed and may represent colonisation or contamination rather than true infection. A significant portion of dermatophyte samples (*n* = 3081) lacked site identification details; most were nail samples (95.2%, *n* = 2932) without differentiation between toe and fingernails. This absence of site information hinders the precision of our analysis. Moreover, a considerable number of samples had incomplete demographic data available, which adds another layer of complexity to interpreting the results. Our study focuses on an area in South‐East England, which may limit how broadly we can apply our findings nationwide. To enhance our understanding of dermatophyte epidemiology on a large scale, future research should gather data from various locations.

In conclusion, our study offers insights into both the clinical and mycological aspects of dermatophyte infections in England spanning 17 years. Studies on the epidemiology of dermatophytosis are of great importance, given the significant increase in recent years of drug‐resistant dermatophytes, including *T. indotineae* and *T. rubrum* . Ongoing surveillance is needed, and robust clinical guidelines are essential to support management of these more challenging infections.

## Author Contributions

Conceptualisation: Khanh Linh Phan, Dr David J. Chandler. Resources: Judith Anne Feeney, Matthew Longbone and all the staff at the Department of Microbiology and Infection, Southeast Public Health Laboratory, Royal Sussex Country Hospital. Investigation: Khanh Linh Phan. Formal Analysis: Khanh Linh Phan, Dr David J. Chandler. Writing: original draft – Khanh Linh Phan. Writing, review and editing: Khanh Linh Phan, Dr David J. Chandler.

## Conflicts of Interest

The authors declare that there are no conflicts of interest related to this manuscript. This study was conducted independently, and the authors have no financial or personal relationships that could influence the outcomes of this research.

## Data Availability

The data that support the findings of this study are available from the corresponding author upon reasonable request.
